# *Chlamydia* in pigs: intriguing bacteria associated with sub-clinical carriage and clinical disease, and with zoonotic potential

**DOI:** 10.3389/fcell.2024.1301892

**Published:** 2024-08-14

**Authors:** Georg Häcker

**Affiliations:** ^1^ Institute of Medical Microbiology and Hygiene, Medical Center, Faculty of Medicine, University of Freiburg, Freiburg, Germany; ^2^ BIOSS Centre for Biological Signalling Studies, University of Freiburg, Freiburg, Germany

**Keywords:** *Chlamydia*, intracellular bacteria, cell biology, immune defence, vaccine

## Abstract

*Chlamydiae* are bacteria that are intriguing and important at the same time. The genus *Chlamydia* encompasses many species of obligate intracellular organisms: they can multiply only inside the cells of their host organism. Many, perhaps most animals have their own specifically adapted chlamydial species. In humans, the clinically most relevant species is *Chlamydia trachomatis*, which has particular importance as an agent of sexually transmitted disease. Pigs are the natural host of *Chlamydia suis* but may also carry *Chlamydia abortus* and *Chlamydia pecorum*. *C. abortus* and possibly *C. suis* have anthropozoonotic potential, which makes them interesting to human medicine, but all three species bring a substantial burden of disease to pigs. The recent availability of genomic sequence comparisons suggests adaptation of chlamydial species to their respective hosts. In cell biological terms, many aspects of all the species seem similar but non-identical: the bacteria mostly replicate within epithelial cells; they are taken up by the host cell in an endosome that they customize to generate a cytosolic vacuole; they have to evade cellular defences and have to organize nutrient transport to the vacuole; finally, they have to organize their release to be able to infect the next cell or the next host. What appears to be very difficult and challenging to achieve, is in fact a greatly successful style of parasitism. I will here attempt to cover some of the aspects of the infection biology of *Chlamydia*, from cell biology to immune defence, epidemiology and possibilities of prevention. I will discuss the pig as a host species and the species known to infect pigs but will in particular draw on the more detailed knowledge that we have on species that infect especially humans.

## *Chlamydia* and *Chlamydia*-like organisms

*Chlamydia* has long been recognized as a bacterial genus whose members cause human infections. *Chlamydia trachomatis* is the cause of genital and eye infections, with a number of strains defined by variations in a surface protein (serovars) having different tissue tropism; serovars D-K are the most common bacterial agents of sexually transmitted disease (Hocking et al., 2023). *Chlamydia pneumoniae* causes airway infections and relatively rarely pneumonia (Wang et al., 2023). *Chlamydia psittaci*, transmitted from birds, is responsible for atypical pneumonia in about 1% of cases (Hogerwerf et al., 2017). Currently, 14 species and four candidate species in the genus *Chlamydia* are known, with hosts as variable as birds, reptiles, cats, ruminants and pigs. In addition, a huge variety of chlamydia-like organisms, or environmental Chlamydiae, have been discovered, which can infect protists such as amoebae and which have often been identified by metagenomics sequencing. Well over 1,000 family-level lineages are likely to exist, presumably with lifestyles related to the ones of the pathogenic chlamydial species. In environmental amoebae, chlamydial species may live as symbionts, providing protection against other bacteria (Konig et al., 2019). Environmental Chlamydiae have been recovered from host populations in the wild, for instance fish and arthropods. They can be found in freshwater, seawater, soil and sediments. The suggestion that there is a Chlamydia for every eukaryote (Collingro et al., 2020) may not be far from the truth. This great success as symbionts and parasites may surprise, because Chlamydiae are obligate intracellular organisms that had to arrange themselves with their host cells. This intracellular life poses a number of challenges, which Chlamydiae have obviously overcome, to co-evolve with their hosts.

## Lifestyle and developmental cycle of *Chlamydia*

Chlamydiae in humans and in pigs (species of the genus *Chlamydia*, family Chlamydiaceae) have a developmental cycle often described as biphasic. The bacteria differentiate between two forms. The elementary body (EB) has very little metabolic activity and is transmitted from one cell to the next, and from one host individual to the other. The EB is taken up by an epithelial cell by endocytosis and rapidly starts altering the vesicle to establish the parasitophorous vacuole that has been termed inclusion (Bastidas et al., 2013). In the inclusion, *Chlamydia* differentiates into reticulate bodies (RBs), which divide (like other bacteria) by binary fission. After about six rounds of division, RBs differentiate back into EBs, possibly triggered by the reduction in size that can be observed in the course of these divisions (Lee et al., 2018). At the end of this developmental cycle, which in cell culture takes about 48–72 h, the EBs are released to infect the next cell (Dautry-Varsat et al., 2004); release may or may not involve lysis of the host cell (Hybiske and Stephens, 2007). The challenges are considerable. *Chlamydia* has to prevent lysosomal fusion of the endosome to prevent its degradation. It has to hide bacterial molecules that can be recognized as foreign by cellular receptors, in order to avoid an immune response. This is not entirely successful: at least bacterial peptidoglycan, which is recognized by the host NOD1 receptor (Buchholz and Stephens, 2006), and chlamydial DNA, recognized by cGAS (Zhang et al., 2014) appear to become accessible and to trigger some inflammatory response in epithelial cells. Apoptosis, i.e., regulated cell death, also appears to be a host defence reaction: environmental Chlamydiae, which are not adapted to mammalian hosts, induce apoptosis in insect cells as well as in human cells, and inhibition of apoptosis allows some development of the bacteria in those cells (Sixt et al., 2012; Brokatzky et al., 2020). Because *Chlamydia* needs the host cell, if the host cell kills itself the bacteria cannot replicate. Chlamydiae adapted to mammalian cells have evolved anti-apoptotic mechanisms and can very potently block experimentally induced mitochondrial apoptosis. A number of different molecular ways have been suggested how this could take place. Our own model is that a chlamydial porin (a channel protein in the outer membrane of *Chlamydia*) can mimic a mitochondrial porin with known anti-apoptotic function (Waguia Kontchou et al., 2022). When *C. trachomatis* is genetically modified in a way that takes away its ability to maintain inclusion integrity, the recognition of bacterial components causes cell death and the bacteria are unable to grow (Sixt et al., 2017). Avoidance of the apoptotic response seems to be one example of what *Chlamydia* has to achieve.

A further problem for *Chlamydia* is nutrient acquisition. The bacteria live inside a vacuole and have to acquire either macromolecules or their building blocks across the membrane. Not only do they have to get hold of all materials for their own replication, they also have to provide for the growing inclusion membrane, which expands during the developmental cycle, when in the end the inclusion can fill most of the cell. *Chlamydia* does all of this without much disruption of the host cell’s physiology. Despite a huge inclusion, cellular functions continue by and large as usual (Moore and Ouellette, 2014). At the end of the developmental cycle, *Chlamydia* faces the problem of organizing its release. It has been suggested that this can occur through lysis of the host cell or by release of the inclusion without lysis, i.e., “extrusion” (Hybiske and Stephens, 2007). As already said, the released elementary bodies have very little metabolism (Omsland et al., 2012). This is a necessary consequence of the chlamydial lifestyle, because EBs lack sophisticated pathways of nutrient acquisition.

*Chlamydia* achieves much of this cellular manipulation and evasion by the secretion of bacterial proteins. There are different types of secretion in bacteria (and in *Chlamydia*) but much of the biological effects of chlamydial infection appears to be achieved by type III secretion. The bacterial type III secretion system (T3S) is a complex protein assembly, looking like and functioning much like a syringe (Galan and Waksman, 2018). About 50 T3S-substrates associate with the chlamydial inclusion membrane (Elwell et al., 2016), and additional proteins are injected into the cytoplasm beyond the vacuole [e.g., Pennini et al. (2010)]; additional proteins are secreted by the bacteria and stay in the inclusion (Gehre et al., 2016). Understanding function and importance of individual secreted chlamydial proteins is a major challenge. Prominent known T3S-effectors include Tarp [an actin-remodelling protein (Clifton et al., 2004)], GlgX and GlgA [glycogen-debranching enzyme/glycogen synthase (Gehre et al., 2016)] and two bacterial deubiquitinases, ChlaDUB1/2 (Misaghi et al., 2006). In addition to their functions in cell biology, secreted chlamydial proteins (T3S-substrates and others) can be immunodominant, such as CPAF (Murthy et al., 2009), Tarp (Wang et al., 2009) and the protein encoded by the chlamydial plasmid, Pgp3 (Donati et al., 2003). These and other proteins can induce a level of protective immunity when included in a vaccine (see section on vaccines, below) and are considered candidates for anti-chlamydial vaccination approaches. It has only relatively recently become possible to modify *Chlamydia* genetically (Kedzior and Bastidas, 2019), and the lack of such tools has slowed down discovery in the past. Only in recent years the deletion or over-expression of individual proteins has become possible, and this is starting to accelerate discovery.

*Chlamydia suis* is very similar to *C. trachomatis*, perhaps reflecting the similar physiology of humans and pigs [indeed, *C. suis* was early on considered a strain or biovar of *C. trachomatis* based on the similarity of the OmpA porin similarity (Schautteet and Vanrompay, 2011)]. Although analysis of *in vitro* growth of *C. suis* has often used mouse or human cells, a study in a pig retinal cell line has investigated its cell biology more closely. This study reported growth characteristics and developmental cycle as well as an inflammatory response (induction of chemokines and cytokines) that were very similar to the one well known for *C. trachomatis* (Kaser et al., 2015). Conversely, when genital epithelial cells from female pigs were infected with *C. trachomatis*, they supported its growth (Guseva et al., 2003). This suggests similarity of the infection of humans with *C. trachomatis* and pigs with *C. suis*.

## Types of chlamydial disease

This is a very wide field. *In vitro*, *Chlamydia* (with differences between species) can infect and grow in multiple cell types. *In vivo*, the infection mostly occurs in epithelial cells in many organs and at many body surfaces. In humans, infections commonly concern the genital tract and the ocular epithelium (*C. trachomatis*) and the airway epithelium (*C. pneumoniae*). *C. trachomatis* is also relatively frequently recovered from rectal swabs, which may indicate intestinal replication (Bavoil et al., 2017). Key features of chlamydial infection of the natural host of a given species are a high share of asymptomatic infection and the potential of long-term carriage. Genital human infection with *C. trachomatis* is asymptomatic in approximately 80% of individuals (Detels et al., 2011). Serological studies suggest that infection rates with *C. pneumoniae* are high during adolescence (Grayston, 1992); although pneumonia may occur, most infections are probably asymptomatic or cause only mild disease (Saikku et al., 1985). Because seropositivity for anti-*C. pneumoniae* antibodies appears to wane quickly after acute infection (Hahn et al., 1991) yet many people remain positive throughout life, infections are either common or remain chronic. Chronic carriage is relatively well documented for *C. trachomatis* infection: with the caveat of re-infection in these observational studies, the half-time of clearance of *C. trachomatis* from the genital tract in women has in two studies been found to be around 1 year (McCormack et al., 1979; Molano et al., 2005). In humans, infection with the avian bacterium *C. psittaci* is known to cause sometimes severe disease (Beeckman and Vanrompay, 2009) although it is often asymptomatic in birds [e.g., Lee et al. (2023)]. While the data are insufficient to be conclusive, they are compatible with the interpretation that transmission of chlamydial species to host species other than their natural host is more likely to cause overt disease. Despite this often-asymptomatic course, chlamydial infection can in a considerable rate of the cases cause substantial tissue damage and severe sequelae. In humans, *C. trachomatis*-infection is a leading cause of female infertility (Zheng et al., 2021). The infection of pigs will be discussed below.

## Immune defence against *Chlamydia*

When *Chlamydia* infects its animal host, the bacteria are recognized as foreign, and a strong immune response ensues. As for most infections, speedy resolution of the infection requires the adaptive immune system, i.e., T lymphocytes with their various functions as well as B lymphocytes. The innate immune system is first in recognizing the bacteria and mounting an inflammatory response, and this inflammation and the immune response are instrumental in the development of tissue damage, a common feature of chlamydial infection. Much of our knowledge of immunity and the immune response to chlamydial infection is derived from studies in small animals, either mice or guinea pigs, with a limited set of bacterial species. Most data are available for infections of mice with *Chlamydia muridarum*, which causes a relatively fast infection of the murine female genital tract. Many activities and requirements for the clearance of chlamydial infection have been established in this model, for instance the requirement for T cells and interferon-γ (Rank et al., 1985). Data on intestinal infection of mice with *C. muridarum* are also accumulating (Zhong, 2021). More recently, protocols for genital infections of mice with *C. trachomatis* have been introduced (this requires trans-cervical infection rather than the human infection through the vagina) (Gondek et al., 2012). It is clear from the multitude of studies that infections with non-natural species of *Chlamydia* will produce results that may not accurately reflect the infection with the naturally occurring species. Although principles are likely to be conserved, the results from mice may have limited relevance for pigs. It is impossible here to review the complete literature on the immune response to *Chlamydia*. The following are however principles that probably apply to chlamydial infections in general.

An initial response is generated by the first infected epithelial cell. A study testing for the transcriptional response in the mouse genital tract very early after infection found expression of numerous chemokines at a very early stage when no infiltrating immune cells were detectable (Rank et al., 2010). *Chlamydia*-infected HeLa cells can produce interleukin-6 and chemokines through the major intracellular signaling pathways (NF-κB, MAP kinases) (Buchholz and Stephens, 2006; Buchholz and Stephens, 2007): the cell detects chlamydial peptidoglycan fragments with the receptor NOD1 (Buchholz and Stephens, 2008), and double-stranded DNA through the sensor cGAS (Zhang et al., 2014). It therefore seems likely that this initial response is triggered by inflammatory stimuli from the directly infected epithelial cells, i.e., non-professional, structural cells, although a contribution by tissue-resident myeloid cells is also possible. There are a number of soluble factors that can modify the immune response, such as anti-microbial peptides (Donati et al., 2005) and the complement system (Megran et al., 1985). As in many infections, neutrophil granulocytes are recruited quickly to the site of infection. Neutrophils have some function in reducing chlamydial burden, as mice with reduced numbers of neutrophils show a slightly reduced efficiency of chlamydial clearance (Zortel et al., 2018).

Chlamydial infection causes tissue and organ damage to varying degrees. The mouse genital infection model with *C. muridarum* has been most closely studied, and damage to the fallopian tubes and infertility are regularly seen in infected mice (Darville et al., 1997). Similar damage is seen in human infection with *C. trachomatis*, and this is indeed the greatest clinical problem of chlamydial infection (Westrom et al., 1992). As is the case for the human infection, chlamydial infection may be asymptomatic or cause disease in pigs (see below). Severe infections may cause enteric damage or abortion, and such infections are associated with damage to the organs.

*In vitro* studies suggest that infected cells will die when the bacteria exit although a non-lytic exit by extrusion of the vacuole has also been proposed (Hybiske and Stephens, 2007). Very likely, most of the tissue damage is caused by the inflammatory and immune response to the infection. Inflammatory responses are very often associated with transient or permanent tissue damage: organ dysfunction is even part of the definition of sepsis, a syndrome driven by hyperinflammation (Singer et al., 2016). Inflammation is mostly the result of the activation of both non-professional tissue cells and cells of the innate immune system, although the mechanisms overlap with the adaptive immune response. Tumour necrosis factor (TNF), for instance, can be secreted by most cells and has been found to contribute to tissue damage during chlamydial infection as an effector molecule of activated T cells (Murthy et al., 2011).

Very clearly, neutrophils make a substantial contribution to tissue damage. When neutrophils were genetically depleted (although some other myeloid cells were also somewhat affected in that model), tissue damage in mice was reduced, and enhancing the life-span of neutrophils by transgenic expression of the anti-apoptotic protein BCL-2 increased tissue damage (Zortel et al., 2018). Intriguingly, the chlamydial plasmid makes a contribution to this damage. Plasmids are extrachromosomal DNA-fragments and often carry non-essential information such as antibiotic-resistance genes. *Chlamydia* has a plasmid that is not required for its *in vitro*-replication but appears to have important functions *in vivo* as basically all clinical isolates are plasmid positive. In murine infection, prior depletion of the plasmid of *C. muridarum* resulted in reduced tissue damage and the establishment of a protective immune response (O’Connell et al., 2007). Although this is incompletely understood, it suggests that virulence and inflammation are linked.

As mentioned above, lymphocytes are required for clearance of the infection. Both T cells (Morrison et al., 1995) and B cells (Su et al., 1997) contribute to protective immunity in animal models, and the same is suggested in humans (Wang et al., 2005). This role of T cells in the immune response to *Chlamydia*–mostly studied in mice–warrants more detailed discussion [for a recent review of this subject matter see (Helble and Starnbach, 2021)]. Both CD4 and CD8 T cells respond to the infection. The evidence is somewhat indirect but it appears that CD8 T cells, whose most prominent function is target cell lysis, are required to clear chlamydial infection not as lytic cells but as producers of IFN-γ (Johansson et al., 1997; Lampe et al., 1998).

CD4 T cells can assume various differentiation states, linked to differential production of cytokines. Chlamydial infection induces not only T_H_1 cells, which can produce IFN-γ, but also T_H_2 and T_H_17 subsets with other cytokine profiles (Zhu, 2018). Their roles during chlamydial infection are not known. In mouse-infection with *C. trachomatis*, CD4 T cells are both necessary and sufficient to clear infection and to protect against re-infection (Gondek et al., 2012). In a T cell receptor transgenic model, the direct activation of T cells in the inguinal lymph nodes and subsequent trafficking to the genital tract was observed, and the T cells produced IFN-γ in the tissue (Poston et al., 2017). The details of chlamydial antigen presentation and recognition are however not well understood. For instance, when the often-used model-antigen ovalbumin was introduced into *Chlamydia*, ovalbumin-specific T cells were stimulated but not protective (Helble and Starnbach, 2019). A relatively recently discovered subset of CD4 T cells, tissue-resident memory cells, remain in non-lymphoid tissue and are relevant for protection against microbial re-infection in many cases (Schenkel and Masopust, 2014). In chlamydial infection in mice, these cells are generated upon mucosal priming and can protect the mice against infection (Stary et al., 2015). It is important to note that mucosal cross-protection has been found numerous times in chlamydial infection models: intranasal priming/vaccination can induce protection at the genital mucosa (Helble and Starnbach, 2021). This appreciation is important for the development of a protective vaccine against chlamydial infection (see below).

## Chlamydial infection in pigs: epidemiology, disease and anthropozoonotic potential

As discussed above, one of the key characteristics of mammalian infection with their “natural” chlamydial species is the wide variation of disease severity, and in particular the high rate of asymptomatic infection. Infection of pigs with *Chlamydia* has high prevalence but in many cases does not cause overt disease. The pig is considered the natural host only of *C. suis*, but *C. abortus* and *C. pecorum* are also isolated from pigs.

### C. suis

*C. suis* has been found in other host species such as sheep (Polkinghorne et al., 2009), cattle (Teankum et al., 2007) and horses (Pantchev et al., 2010). The anthopozoonotic potential is suggested by the, albeit rare, detection of *C. suis* in trachoma patients (Dean et al., 2013) and in ocular, nasal or pharyngeal swabs as well as from fecal samples in abattoir workers and pig farmers (De Puysseleyr et al., 2014; De Puysseleyr et al., 2017). There appears to be a very high prevalence in pigs: one study reported over 90% positivity of fattening pigs in fecal samples and around 50% in conjunctival swabs in Swiss farms (Hoffmann et al., 2015). A Chinese study reported positivity of 62.4% of rectal samples (Li et al., 2017). The rate of disease among pigs carrying *C. suis* is difficult to pin down. Symptoms and infections such as rhinitis, conjunctivitis, enteritis, pneumonia and reproductive disorders have been scribed to *C. suis* infection, as has been deterioration of semen quality [reviewed in (Schautteet and Vanrompay, 2011)]. It has been suggested that *C. suis*-infection can contribute to conjunctivitis in both domestic pigs (Chahota et al., 2018) and wild boars (Risco et al., 2013), as well as to enteritis and persistent shedding (Hoffmann et al., 2015). Although a connection between *C. suis*-infection and reproductive problems has also been proposed (Kauffold et al., 2006), the majority of in particular enteric infections appears to be asymptomatic (Li et al., 2017).

### C. abortus

*C. abortus* is primarily a sheep pathogen and is responsible for a considerable number of abortions in these animals (Longbottom and Coulter, 2003). Abortion here is the consequence of placental infection with *Chlamydia* (Livingstone et al., 2017) although, as is the case for other species, chlamydial carriage is often asymptomatic (Lenzko et al., 2011). Abortion in swine due to *C. abortus*-infection does not appear to be very frequent but has been described (Salinas et al., 2012).

### C. pecorum

*C. pecorum* is a frequent agent of chlamydial infection in ruminants and in swine. Infections can cause a wide spectrum of disease, such as conjunctivitis, enteritis, abortion, polyarthritis, pneumonia and urogenital disease (Mohamad and Rodolakis, 2010; Reinhold et al., 2011). In pigs, *C. pecorum* is typically found together with *C. suis*, with the incidence of *C. pecorum* increasing towards the end of the fattening period (Hoffmann et al., 2015). Even in cattle and sheep, the role of *C. pecorum* has been suggested to be under-recognized (Walker et al., 2015). It is probably fair to say that not enough information is available to assess the importance of carriage and infection of swine with this chlamydial species.

Taken together, the clinical features of chlamydial infection in swine seem to be very similar to the known pattern from humans. Carriage may be prolonged and is mostly asymptomatic, but disease is common enough to be of considerable importance.

## Treatment of infection

True, acquired antibiotic resistance is rare in *Chlamydia*, and antibiotic therapy of chlamydial infection is therefore relatively straightforward. The notable exception is the tetracycline-resistance in *C. suis*. This resistance is due to the chromosomal integration of a resistance cassette, which likely predates the clinical use of tetracycline but whose presence in *C. suis*-strains has probably been selected for by the use of tetracycline in animal feed and in treatment (Hoffmann et al., 2015; Joseph et al., 2016). The resistance cassette can experimentally be transmitted from *C. suis* to *C. trachomatis* and *C. muridarum* by co-infection in cell culture (Suchland et al., 2009) but such transfer has not been documented naturally. Tetracyclines are commonly used to treat infections, and this resistance is therefore relevant. However, the tetracycline-resistant strains retain sensitivity to other antibiotics such as rifamixine and fluoroquinolones (Borel et al., 2016). The quinolone enrofloxacine is considered an alternative for infections with tetracycline-resistant strains; macrolides (erythromycin) can also be considered (Schautteet and Vanrompay, 2011). Despite the documented or likely sensitivity to the antibiotics used, treatment has failed to eradicate Chlamydiae at the herd level in a Swiss study (Hoffmann et al., 2015).

## Anti-chlamydial vaccines

As is the case for any infectious disease, preventing chlamydial infection by a vaccine may have very substantial benefit. Even though it is at this stage difficult to quantify this benefit in pigs, if a vaccine were available it should certainly be tested in herds of swine. Most, perhaps all clinically relevant infections with any pathogen cause an immune response, which typically results in some level of immunity. Human genital infection with *C. trachomatis* is cleared with greatly varying speed, and a correlation between time to clearance and reinfection has been noted in patients (Geisler et al., 2013). *C. muridarum*-infection of mice causes substantial if incomplete protection against reinfection (De Clercq et al., 2013). Very intensive research into anti-chlamydial vaccines for human use has been underway for decades but no vaccine is close to clinical use. Indeed, despite many studies, the immunological correlate of protection against infection is still not very well defined: do we need antibodies, or interferon-producing or cytotoxic T cells that recognize *Chlamydia* and *Chlamydia*-infected cells? Which antigens are best? In the 1960s, a number of human trials have been conducted against trachoma, a severe eye-infection with *C. trachomatis* (the first study was already initiated in 1913) (de la Maza et al., 2017). Immunodominant chlamydial proteins such as Tarp (Wang et al., 2009), Pgp3 (Peng et al., 2022) and CPAF (Murthy et al., 2006) can induce some protective immunity against chlamydial infection. However, none of these approaches has completely prevented the infection; the benefit of such vaccines is therefore difficult to predict.

A 2019 study showed antigenicity of a *C. trachomatis* vaccine preparation in humans (Abraham et al., 2019). It seems clear that it is still a long way to go before a vaccine–which is particularly desirable for protection against genital infection–becomes available for human use. No vaccine against *C. suis* infection is available either. *C. abortus*-infection is more relevant in ruminants than in pigs, and two whole-cell vaccine preparations are available for the use in sheep, where they prevent abortion and reduce bacterial shedding (Garcia de la Fuente et al., 2004; Rocchi et al., 2009). An experimental subunit vaccine against *C. abortus* has shown immunogenicity in piglets (Ou et al., 2013), and similar immunogenicity has been found against *C. pecorum* in the endeavour to prevent disease in koalas (Desclozeaux et al., 2017).

There are a number of vaccination efforts against chlamydial infection in pigs. Most of these studies have used pigs as model organisms to evaluate vaccines against *C. trachomatis*-infection [for instance (Vanrompay et al., 2005)]. *C. trachomatis* causes infection and tissue inflammation in minipigs (Erneholm et al., 2016), and indeed, more recent studies reported protective effects with either UV-irradiated *C. trachomatis* EBs or an adjuvant-supplemented chlamydial protein against infection with *C. trachomatis* in minipigs (Boje et al., 2016; Erneholm et al., 2019). Recent studies into a *C. suis*-vaccine for pigs are encouraging. The lymphocyte, especially T cell response of pigs to this pathogen has been studied in detail, and multi-functional T cells were induced by the infection (Kaser et al., 2017). Recently, a protein vaccine (CPAF protein from *C. trachomatis*) has been found to be very immunogenic in pigs (Proctor et al., 2024). It has further been reported that UV-inactivated *C. suis*-EBs were able to induce IFN-γ-producing T cells and reduce the bacterial load in experimental infection of pigs (Amaral et al., 2020). Perhaps that was not unexpected given the protection seen by this type of vaccine in other models but it is clearly an important and encouraging observation. One study tested a whole-cell vaccine of *C. abortus* in sows and reported immunogenicity although protection was not tested (Knitz et al., 2003).

Given the wide prevalence of *Chlamydia* in pig herds and the incomplete protection provided by available vaccines against species of *Chlamydia*, it is unlikely that a vaccine will be able to eradicate the organisms even from domestic pigs. Whether a vaccine will be able to prevent or alleviate clinical disease is speculative at this stage.

## Concluding remarks

Bacteria of the genus *Chlamydia* (and at least to some extent of the order *Chlamydiales*) are amazingly well adapted, cell biologically to their host cell and also in multicellular hosts to the host’s immune system. Symptoms of infection and carriage are not as a rule clinically apparent. However, the high infection rate in pigs by *C. suis* and carriage of other species make it a relevant pathogen. Other than the curious tetracycline-resistance of *C. suis*, antibiotic resistance is not a major problem in chlamydial infections. Effective vaccines are still lacking, but it seems possible that a relevant protection against infection can be achieved. Chlamydial elimination from domestic pigs is not realistic. However, we do have the test systems to understand the epidemiology, and there are ways of controlling the infection.
